# Applying a co-designed medication plan for safer medication treatment in older persons: a feasibility study

**DOI:** 10.1186/s40814-025-01661-1

**Published:** 2025-07-03

**Authors:** Malin Holmqvist, Axel Ros, Johan Thor, Linda Johansson

**Affiliations:** 1 Department of Public Health and Healthcare, Region Jönköping County, Jönköping, Sweden; 2 Futurum, Region Jönköping County, Jönköping, Sweden; 3 Jönköping Academy for Improvement of Health and Welfare, School of Health and Welfare, Jönköping University, Jönköping, Sweden; 4 Institute of Gerontology, School of Health and Welfare, Jönköping University, Jönköping, Sweden

**Keywords:** Feasibility, Medications, Older people, Patient safety, Usability

## Abstract

**Background:**

To promote patient safety, international guidelines highlight the importance of a joint plan for continued treatment in older persons. Accordingly, in a co-design initiative involving older persons and healthcare professionals, a medication plan was developed. This study aimed to assess the feasibility of applying the medication plan for older persons in primary care. The objectives were to examine the feasibility of the medication plan in clinical practice and to examine the feasibility of methods that could be used to study a broader implementation.

**Methods:**

A prospective study design, using both qualitative and quantitative methods, was employed. During appointments at primary care centres, physicians (*n* = 6), persons aged 75 or older (*n* = 21), and, when applicable, their next of kin (*n* = 2) collaboratively agreed on a medication plan, which was documented in the electronic health record. Over a 3-month follow-up period, data regarding the feasibility of the medication plan (usability and fidelity) and the feasibility of the research methods (recruitment and retention rates, data collection, and outcome measures) were collected.

**Results:**

Usability assessed by the System Usability Scale scored a median of 51.3 out of 100 for physicians. The participants’ experiences of the medication plan’s usability addressed functionalities, individualisation for relevance, resources, and a de-prioritised medication plan. Fidelity was assessed based on 8 out of 15 older persons reporting that they had received a medication plan, and 59% of all prescribed medications had documented goals and/or comments. The recruitment rate was 75% for physicians and 70% for older persons. There were no changes in polypharmacy and no contact with healthcare due to suspected adverse drug events. The participants’ perceptions of the medication plan’s ability to promote patient safety addressed awareness and information, challenges beyond the medication plan, and patient involvement.

**Conclusions:**

The implementation of the co-designed medication plan encountered challenges related to usability and fidelity, requiring collaborative refinements of the prototype. Additional difficulties arose from using a low-fidelity prototype in clinical practice. Our results emphasise the strength of combining qualitative and quantitative methods to capture participants’ perspectives on the medication plan’s ability to promote patient safety. Before conducting a larger study, the evaluation methods require further refinement.

**Trial registration.:**

ClinicalTrials.gov NCT06016140 (retrospectively registered).

**Supplementary Information:**

The online version contains supplementary material available at 10.1186/s40814-025-01661-1.

## Key messages regarding feasibility

- *What uncertainties existed regarding the feasibility?* Key uncertainties concerned both the feasibility of the medication plan and the research methods. Before evaluating usability in a larger study, the medication plan needs to be further developed to meet functional requirements.
- *What are the key feasibility findings?* The intervention met challenges related to both usability and fidelity, highlighting the difficulties of using a low-fidelity prototype in clinical practice. Moreover, appropriate tools to measure patient safety from the patient perspective in a Swedish primary care context need to be further developed and validated.
- *What are the implications of the feasibility findings for the design of the main study?* To align with resilience thinking, an individualised intervention based on the older person’s perceptions of having a medication plan may represent a more appropriate study design for a larger study.

## Background

Older persons are at a higher risk of harm from medications, and adverse drug events tend to occur when a medication is prescribed or not re-evaluated [1, 2]. To promote patient safety for older persons, it is essential to design interventions that specifically target these areas. One approach, which has been explored to a lesser extent, is to design interventions collaboratively with older persons and subsequently evaluate their outcomes.

When asking older persons and healthcare professionals about their experiences of evaluation of medications, the older persons want to be involved in the evaluation but need more knowledge and written information about the plan for monitoring and evaluation [3]. Nurses and physicians in primary care experience that the evaluation of older persons’ medications is influenced by healthcare professionals’ clinical knowledge, experiences, and situational conditions, and that cooperation with and around the older person is central [4]. International guidelines point to the value, for both older persons and healthcare professionals, of a joint plan for continued treatment to promote patient safety [5]. Safer medication treatment may be promoted when healthcare professionals collaborate with older persons to formulate goals for medications and describe procedures for monitoring and evaluation [6–8]. Nevertheless, there is not enough knowledge or evidence for such an approach, although emerging evidence suggests that empowered patient involvement and patient safety may be interrelated. Patients, as co-creators of resilience, can positively influence outcomes by managing unwanted variability within healthcare [9].

When shaping and improving healthcare, patients, their next of kin, and healthcare professionals should be consulted and involved in the design of new services [10]. A co-design approach can increase the acceptability and integration of an intervention in clinical practice by accommodating problems identified by stakeholders in the development process. To ensure that a joint plan for continued treatment fulfils the involved persons’ requests, a medication plan was co-designed by older persons and healthcare professionals [11]. The co-design process followed the two middle phases of the Double Diamond framework’s four phases — discover, define, develop, and deliver [12]. In the last phase, deliver, the co-designed service or product is supposed to be launched and tested, and needs and requirements identified in the previous phases are addressed through feedback from participants. Subsequently, older persons, their next of kin, and healthcare professionals should test the co-designed medication plan in clinical practice. This approach can be seen as a complex intervention as it contains several interacting components, targets different groups or levels, and has different degrees of flexibility and variability of outcomes [13]. Before conducting large-scale evaluations of a complex intervention, assessing the feasibility of the intervention can provide diverse perspectives on the process, supporting decisions regarding further evaluation and implementation [13]. A feasibility study approach is valuable for assessing an intervention and for evaluating a study design and potential clinical outcomes [13–15] and was used to assess whether a co-designed medication plan for older persons was suitable for further evaluation.

### Objectives

The overall aim was to assess the feasibility of applying a medication plan for older persons in primary care. The objectives were as follows:

- Examine the feasibility of a medication plan in clinical practice
- Examine the feasibility of the research methods that could be used to study a broader implementation.

## Methods

### Study design

A non-randomised, prospective, single-arm design was used to apply the co-designed medication plan for persons aged 75 or older in primary care. No changes in the design were made after the study began. The first author followed the medication plan for each older person for 3 months. The period was chosen for pragmatic reasons, with the primary aim of examining the medication plan’s feasibility, still recognising that medications are often re-evaluated yearly. The study is reported according to the Consolidated Standards of Reporting Trials (CONSORT) statement: extension for pilot and feasibility trials [16, 17] (Appendix 1).

### Sample size

While formal sample size calculations are not required for feasibility studies [18], it remains important to provide a clear justification for the chosen sample size. This study aimed to include 30 participants, in line with general recommendations for pilot studies, which suggest that such a sample is sufficient to estimate feasibility parameters [19]. Additionally, a British audit of 79 pilot and feasibility studies reported a median sample size of 30 participants per arm (range 8 to 300) [18], further supporting the appropriateness of the selected number.

### Setting and participants

The study was conducted at primary care centres in one public healthcare region in southern Sweden, serving 369,000 citizens in 2022, where individuals aged > 75 years constituted 11% of the total population [20]. Recruitment took place from June 2022 to May 2023.

To achieve a total of 30 participants and obtain a variety of perspectives, the objective was to recruit 10 physicians, with each physician cocreating a medication plan with 5 older persons. First, physicians were asked to participate through a general invitation from the first author, sent by e-mail to all managers at 42 primary care centres in the healthcare region. The inclusion criteria for physicians were as follows: being employed at a primary care centre and having regular job-related encounters with older persons. Older persons were invited to take part by the participating physician or a nurse working at the primary care centre where the older person was registered. The inclusion criteria for older persons were as follows: being at least 75 years old, using five or more medications, and having the ability to understand and communicate in Swedish and scheduled for an appointment with one of the participating physicians. Exclusion criteria were as follows: a documented diagnosis of dementia and in the late palliative phase, defined as an estimated life expectancy shorter than 6 months. Persons who supported the older persons (next of kin or nurse) with medications were invited to participate in the study if the older persons agreed to have them invited. Inclusion criteria for supporting nurses were as follows: regular contact with the older person regarding medications and employment at a primary care centre or in municipality-based home healthcare. Inclusion criteria for next of kin were as follows: being at least 18 years old, regular contact with the older person regarding medications, and having the ability to understand and communicate in Swedish.

Demographic profiles of the participants were registered at inclusion. For the older persons, age, sex, the number of medications at inclusion, and whether they managed their medication by themselves were registered. For physicians and nurses, sex and the number of years as a registered physician or nurse were registered. For next of kin, sex, age, and relation to the older person were registered.

### Intervention

Before the appointment, the older persons and physicians received both written and verbal information about the intervention. In addition, each physician received a written guide for documenting in the medication plan template. Although no explicit training was provided, physicians could contact the first author for additional verbal information if needed. The intervention consisted of the physician and the older person jointly agreeing on a plan for continued medication treatment during a scheduled appointment at the primary care centre. The physicians were asked to document the medication plan during the appointment and to update and redistribute it if medications were changed (added, withdrawn, or changed in dose) during the study period. At the set appointment, information about medications, treatment indication, treatment goal, the next step in treatment, what to monitor and when, and who was responsible for what regarding the medications was to be documented in the medication plan template [11, 21] within the older person’s electronic health record (EHR). The older person was to receive the documented plan as a paper printout. If someone (nurse, next of kin) supported the older person with their medications, that person was to receive a paper printout of the medication plan as well. The extra time to apply the medication plan was estimated by the authors to be 10 min.

### Progression criteria

Predefined progression criteria are commonly used in feasibility studies to guide decisions about whether to proceed to larger trials [17]. However, there remains limited guidance on how to establish such criteria in feasibility studies [22]. Instead, in this study, the feasibility was assessed by a comprehensive evaluation of key areas, including recruitment, the suitability of the research methods, data collection procedures, and the acceptability of the intervention.

### Outcome measures

The feasibility of the medication plan and the feasibility of the research methods were examined as outcome measures (Table 1).

**Table 1:** Summary of domain, outcomes, measures and methods of analysis

Outcomes of the feasibility study			
**Domain**	**Outcome**	**Data collection**	**Analysis method**
***Feasibility of the medication plan***	Usability	System Usability Scale (questionnaire)	Descriptive statistics
		Experiences related to usability (interviews with semi-structured questions)	Content analysis
	Fidelity	Number of medications with added information (EHR review)	Descriptive statistics
		Number of older persons reported to have received a medication plan (one question in the questionnaire)	Descriptive statistics
***Feasibility of the research methods***	Recruitment	Percentage of eligible participants that enrolled in the study	Descriptive statistics
	Retention	Percentage of enrolled older persons who completed the intervention	Descriptive statistics
	Data collection procedure	Ease of data collection (in interviews and questionnaires)	Descriptive statistics
		Response rate to questions (to questions in the questionnaire)	Descriptive statistics
	Outcome measures	Experiences of patient safety (12 questions from the National Patient Survey)	Descriptive statistics
		Perceptions related to patient safety (interviews with semi-structured questions)	Content analysis
		Polypharmacy (EHR review)	Descriptive statistics
		Utilisation of healthcare due to suspected adverse drug events (EHR review)	Descriptive statistics

#### Feasibility of the medication plan

The *usability* of the medication plan was assessed with “the System Usability Scale” (SUS) [23–25]. A Swedish version of the SUS [26] was used (Appendix 2). The SUS consists of 10 questions answered on a Likert scale (1 to 5). Each question’s raw score is recalculated to a new value ranging from 0 to 4. The sum of these values is then multiplied by 2.5 to yield an overall score ranging from 0 to 100. An average SUS score above 70 is considered to indicate acceptable usability [23]. Experiences related to the usability of the medication plan were additionally explored by interviews, using an interview guide containing semi-structured questions guided by topics addressed in the SUS [24] (Appendix 3).

*Fidelity* may help in evaluating the study process and determining why the intervention works or fails unexpectedly or yields unexpected consequences [13]. Fidelity was assessed by the number and group of medications (according to the Anatomical Therapeutic Chemical (ATC) classification [27]) with added information collected from the EHRs.

Additionally, the number of older persons who reported having received a medication plan was assessed by one question in the older persons’ questionnaire.

#### Feasibility of the research methods

*Recruitment* was assessed as the percentage of eligible participants enrolled in the study. Eligible participants were those who met the inclusion (and not the exclusion) criteria, had received information about the study, and allowed the first author to provide them with further information. Due to the recruitment strategy, where the physicians were asked to participate through their managers at the primary care centres and participating physicians or nurses working at the primary care centre invited the older persons, the number of potentially eligible participants cannot be counted.

*Retention* was assessed as the percentage of enrolled older persons who completed the intervention, i.e. who had at least one documented medication plan in their EHR.

*Data collection procedures* were assessed as the ease of data collection, using the number of participants answering one or more questions in the questionnaire and the number of participants in the interviews. The response rate to questions in the questionnaire was assessed as the proportion of responses to questions in the questionnaire.

*Outcome measures were as follows*: To evaluate the older persons’ experiences of patient safety in terms of involvement, information, and continuity, 12 questions from the Swedish National Patient Survey Primary Care 2022 [28] were chosen (Table 3), with answers on a 5-grade Likert scale (1-5). Outcomes are presented as the percentage of positive responses, where the numerator consists of those who have responded positively (4 and 5) and the denominator consists of those who responded to the question (1 to 5). Perceptions of patient safety were additionally explored by interviews using an interview guide containing semi-structured questions guided by the 12 used questions from the Swedish National Patient Survey Primary Care [28] and with inspiration from indicators for safety monitoring in healthcare [29] (Appendix 3). Polypharmacy was assessed using the number of medications prescribed in the medication list within the EHR after the appointment in which the medication plan was applied and then again after 3 months. Utilisation of healthcare was assessed using the number and cause of unplanned documented visits or contacts to a nurse or physician within the healthcare region due to suspected adverse drug events during the follow-up period.

### Data collection

The first author collected data on the number of medications, the information documented in the medication plans, and the number and causes of healthcare visits from EHRs.

All older persons and persons supporting them were invited to share their experiences of the medication plan after 3 months. Physicians were invited similarly 1 month after documenting their last medication plan. Physicians received an e-mail containing a link to a web questionnaire. In addition to the SUS questions, the questionnaire included two general questions about how often they had used the medication plan and whether they knew if their patients had used it. It also included one open-ended question for them to provide additional reflections. The older persons and persons supporting them received a questionnaire by mail, with the option to respond via a web link. In addition to the SUS questions and questions from the National Patient Survey, the older persons were asked three general questions to determine: whether they had received a medication plan, if they had used it, and whether their next of kin or healthcare professionals had used it. They were also asked an open-ended question to provide additional reflections. If they reported not having received a medication plan, they were not required to answer additional questions. The next of kin received a similar questionnaire, which included the SUS questions, three general questions similar to those posed to the older person, and an open-ended question to provide additional reflections. Similarly, if they reported not having used the medication plan, they did not have to answer additional questions. All participants received one reminder to complete the questionnaire.

Furthermore, all participants received an invitation to participate in one remote interview, conducted via Skype or telephone. The physicians replied via e-mail if they wanted to participate, and a meeting was scheduled. The older persons and next of kin were contacted approximately 1 week after the mail was sent and asked to participate. If they agreed to participate, the interview was either conducted immediately or scheduled for later. If there was no response after three phone call attempts, the interview was considered declined.

The interviews, conducted individually or in pairs, were performed by the first author, were audio-recorded, transcribed verbatim, and lasted between 8 and 34 (median 15) min.

### Data analysis

The study sample was small (*n* = 21); therefore, descriptive statistics, encompassing mean, median, and percentage calculations, were used to analyse quantitative data.

The interview transcripts were analysed by the first and last authors with qualitative inductive content analysis in accordance with Elo and Kyngäs [30]. To support the analysis, data were entered into NVivo software (QSR International). Open coding was used, which involved marking headings relevant to the area of analysis (usability and patient safety) in the transcripts. Headings with similar content were compared and grouped to generate subcategories. By abstraction, similar subcategories were formed into generic categories and finally main categories reflecting the interviews’ content. Preliminary results were discussed and refined within the author group.

### Ethics declarations

The study adhered to the Declaration of Helsinki [31] and was approved by the Swedish Ethical Review Authority (dnr 2022–04430-01). Providing documentation in the EHR regarding prescribed medication is already mandatory in Sweden [32] and is therefore not considered a new task. Before contact with the first author, the older persons received brief verbal information about the study from staff at the primary care centre and were asked if the first author could contact them about the study. Before inviting persons (next of kin, nurses) who supported a participating older person with medications, the older person was asked for permission to do so. All participants received written information about the study and provided written consent before the intervention and data collection started. To maintain confidentiality, data were de-identified by allocating an ID number to each participant instead of using their names. Data are presented so that no single individual can be identified and are kept secure by the entity responsible for the research following national and local routines.

## Results

### Demographic profiles and baseline data of the participants

Data were collected between November 2022 and July 2023. The baseline characteristics for the participants are presented in Table 2.

**Table 2:** Demographic profile and baseline data of the participants (the number of persons is presented if not stated otherwise)

Participants (*n*)	Items	
Older persons (21)	Sex	
	• Women	11
	• Men	10
	Age (mean and range)	84 (76–97)
	Number of medications at inclusion (median and range)	Regular 9 (5-15)
		As needed 2 (0–12)
		In total 11 (5-24)
	Manage medications with support	
	• Yes	4*
	• No	17
Physicians (6)	Sex	
	• Women	4
	• Men	2
	Years as a registered physician (mean and range)	17 (10–32)
Persons supporting older persons (2)	Sex	
	• Women	2
	• Men	0
	Age (mean and range)	81 (74–88)
	Relationship	
	• Next of kin	2**
	• Nurse	0

^*^Two older persons agreed to invite their next of kin. **Supporting next of kin: daughter (1) and wife (1)

### Feasibility of the medication plan

#### Usability

From the *SUS*, the physicians’ (*n* = 6) median response value was 51.3 (range 22.5–67.5), the older persons (*n* = 4) had a median value of 75.0 (range 50.0–100.0), and the next of kin (*n* = 1) had a value of 80.0.

The analysis of the interviews described the participants’ *experiences of the usability of the medication plan in clinical practice* and is presented in four generic categories: De-prioritised, functionalities, individualisation for relevance, and resources, with associated subcategories and illustrative quotes. These are presented in Appendix 4 and are elaborated further below:

- *De-prioritised.* Other means than the medication plan were applied so the older person could have information about medications, and medical notes were the physicians’ first choice for medical information:
  - ◦ *Older persons use medication lists diversely: *Optimal methods to provide information vary, and handwritten or verbal information may be seen as less burdensome. Older persons tend to use the printed list of prescriptions provided by the pharmacy, overlooking the medication list from healthcare. Individuals with multiple-dose dispensing receive another medication list.
  - ◦ *Physicians use medical notes for information*: Physicians consult medical notes within EHR for planning and treatment history, making it necessary to document plans in both medical notes for the physician and in the medication list for the older person. This results in duplicated documentation, with the medical notes being the preferred choice for physicians.
- *Functionalities.* The older persons did not always recognize that they had received a medication plan, and while the application of the plan within the EHR was technically challenging, it provided a structured approach for information provision:
  - ◦ *A visible, clear structure: *Incorporating the medication plan into the medication list results in a clearer and visible structure, facilitating oversight of relevant information. The structured format within the digital medication plan not only enhances systematic documentation but also compels physicians to thoughtfully plan and effectively communicate plans for continued treatment.
  - ◦ *Limited by technical constraints: *The design of the medication plan is constrained by limitations in available and mandatory fields within the EHR, potentially complicating the process of effectively communicating essential information. These technical challenges demand effort and time, place a significant burden on physicians, and may lead to stressful situations. Technical issues are additionally challenging for older persons.
  - ◦ *Older persons do not comprehend the medication plan: *The older persons struggled to grasp the medication plan due to ambiguity concerning whether the medication list encompassed the plan or due to a misunderstanding of the term “medication plan”. Furthermore, the constant influx of paperwork from healthcare services left older persons unable to keep track of the plan.
- *Individualisation for relevance.* The medication plan was not always considered relevant, related to the older persons themselves or to a specific medication:
  - ◦ *Adapt for individuals:* Adapting the medication plan for older persons is crucial, taking into account those who are willing and able to participate in evaluation of their medications. Achieving a balance of information density presents challenges, given the varying preferences of both healthcare professionals and older persons. Having insufficient information risks hindering comprehension, while excessive detail impairs readability.
  - ◦ *Adapt for medications*: Applying a medication plan was perceived as favourable for newly initiated medications, as it facilitates thoughtful considerations about the treatment plan. It seemed unnecessary to have a plan for medications that have been used for a long time. Additionally, comprehensive planning is not feasible for medications with indistinct therapeutic goals, such as topical agents.
- *Resources.* Benefits need to compensate for the negative impact on resources, and the medication plan was time-consuming to apply:
  - ◦ *Balancing time and purpose*: A medication plan poses questions of resource allocation for physicians and how time should be prioritised, as applying the plan takes time away from other essential tasks, such as proper examination of the patient. The medication plan is valuable and beneficial if sufficient time is available within the daily workload. If the older person recognises its importance, the effort expended on it becomes worthwhile.
  - ◦ *Time-intensive application*: Applying the medication plan during appointments is time-consuming, as the documentation of the medication plan demands a substantial investment of time. A thorough medication reconciliation with older persons, often involving an extensive medication list, requires a significant amount of time, followed by additional time for treatment planning.

#### Fidelity

Of all prescribed medications (*n* = 267), 59% had treatment goals and/or comments about the continued plan documented in the medication plan. Medications taken regularly (*n* = 196) had treatment goals and/or comments documented in 69% of cases, whereas 31% of medications prescribed “as needed” (*n* = 71) had goals and/or comments documented. Medications initiated during the study period (*n* = 24) had goals and/or comments documented in 4% of cases. Treatment goals and/or comments documented in the medication plan varied between the conditions they were prescribed. For instance, medications prescribed regularly for the cardiovascular system (ATC-code C) (*n* = 67) had documentation in 94% of cases, medications used for diabetes (ATC-code A10) (*n* = 13) had 85% documentation, and medication for the nervous system (ATC-code N) (*n* = 17) had documentation in 47% of cases.

According to the responses to the first question in the questionnaire for the older persons, 8 out of 15 respondents reported that they had *received a medication plan* during the appointment.

### Feasibility of the research methods

#### Recruitment and retention

Two of eight eligible physicians declined participation due to lack of time; thus, the *recruitment rate* was 75%. After receiving written and verbal information about the study, 10 of the 33 eligible older persons declined participation, resulting in a *recruitment rate* of 70% (23/33). Four older persons received support with their medications from next of kin. Two of the older persons agreed to let the first author contact them, and both next of kin agreed to participate.

**Fig. 1:**
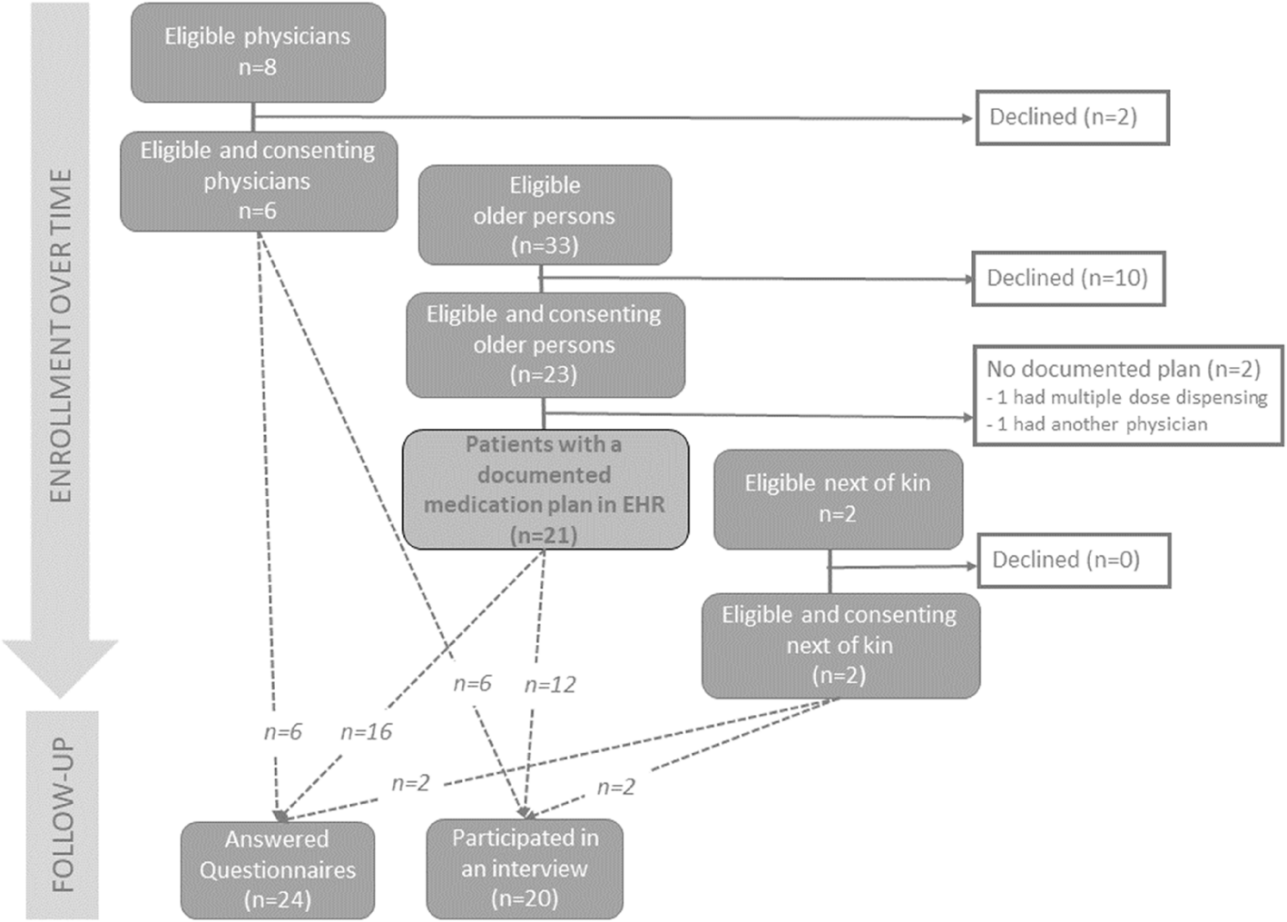
Flow chart with recruitment and retention

Twenty-one of the 23 recruited older persons had a documented medication plan in their EHR after the appointment, resulting in a *retention rate* of 91% (see flow chart in Fig. 1).

#### Data collection procedure

*Ease of data collection* was assessed for both the interview and questionnaire. With one reminder, 24 of 29 participants answered one or more questions in the questionnaire with varying response rates in the three groups: all physicians *n* = 6 and next of kin *n* = 2, whereas among the older persons, 16 of 21 answered. Furthermore, 20 of 29 participants agreed to participate in an interview. All physicians (*n* = 6) and next of kin (*n* = 2) agreed. Of the older persons, nine (43%) did not participate. The reasons were lack of energy (*n* = 4), not recognising that they had received a medication plan (*n* = 3) or not answering the phone call (*n* = 2).

*Response rates to questions* were assessed for the questionnaire. Sixteen participants answered 1 or more of the SUS questions, but to calculate a value for SUS, all 10 questions must be answered. Thus, 11 participants (67%) had a valid score: physicians (*n* = 6), older persons (*n* = 4) and next of kin (*n* = 1). In the questions about patient safety, 10 of 21 older persons (48%) answered one or more questions (Table 3).

**Table 3:** The number of positive respondents for each question in the Swedish National Patient Survey Primary Care

Question and number of respondents	Number of positive respondents *
Were you involved in decisions about your care/treatment to the extent you wanted? (*n* = 10)	8
Did the doctor involve you in decisions about your care/treatment? (*n* = 10)	8
Did the doctor take into account your own experience of your illness/health condition? (*n* = 10)	8
At your visit, was a decision made about the next step in your care/treatment? (*n* = 7)	5
If you spoke with several staff members during your visit, were they consistent in their communication? (*n* = 5)	3
Were you given enough information about medications and possible side effects? (*n* = 10)	5
Did you receive enough information about warning signals to be aware of regarding your illness/health condition or medication treatment? (*n* = 7)	3
Did you receive enough information about your care/treatment? (*n* = 10)	8
Did the doctor explain the medication/treatment in a way that you understood? (*n* = 10)	8
If you asked the staff questions, did you get answers that you understood? (*n* = 6)	5
Were you given the opportunity to ask the questions you wanted? (*n* = 10)	9
If your family/next of kin wanted to talk to a doctor, were they able to do so? (*n* = 1)	0

^*^A positive response is defined as an answer of 4 or 5 on the Likert scale

#### Outcome measures

The older persons’ *experiences of patient safety* in terms of involvement, information, and continuity, assessed by questions from the Swedish National Patient Survey Primary Care (Table 3), had a lower proportion of positive responses compared to national outcome data [28], except for the question about being given the opportunity to ask the questions they wanted. Due to the low response rate (*n* = 10), no conclusions can be made regarding the outcome.

The analysis of the interviews described the participants’ *perceptions of the medication plan’s ability to promote patient safety* and is presented in three generic categories: Awareness and information, challenges beyond the medication plan, and patient involvement, with associated subcategories and illustrative quotes. These are presented in Appendix 4 and are elaborated further below:

- *Awareness and information*: The medication plan contributes to and encourages engagement but may cause concerns. Written information about medications offers clarity and is a means of communicating with the older person, next of kin, and nurses as follows:
  o *Information as a source of reassurance*: The information within the medication plan contributes to a sense of control among older persons and their next of kin, as it provides details about their medications. The medication plan can encourage engagement and generate a feeling of reassurance. However, at times, the information might result in additional confusion and anxiety, especially if someone feels uncertain or there is an excessive focus on clinical hard values.
  o *Provides information about ongoing treatment*: The medication plan provides accessible written information at home through either a printout or the EHR, promoting clarity. The information in the medication plan informs older persons and healthcare professionals of the purpose of treatment, articulates intended treatment goals (such as target blood pressure or blood sugar levels) and potential side effects to monitor, and offers insights into scheduling future follow-ups.
  o *Supports communication*: Communication about medication, such as when conducting a medication reconciliation, is essential when applying the medication plan, as it helps identify medication-related problems. The medication plan is a valuable means of communicating with next of kin and nurses in home healthcare if the older person is unaware of what has been discussed during their appointment.
- *Challenges beyond the medication plan*: Applying a medication plan is difficult due to the complexity of medication use for older persons. There is a risk that older persons can adopt a passive approach and trust in the healthcare services to take proper actions as follows:
  o *Complexity in older persons’ medication treatment*: Formulating goals can be challenging, particularly among older persons, given that much of their treatment focus is on prevention or relief rather than cure. The absence of detailed information about medication in the medical records further complicates this process. Assessing the necessity of a medication for an older person can prove complex, as effectiveness in comparison to other factors such as side effects or normal ageing can be difficult to judge. The complexity can cause a continuation of medications that are not needed.
  o *Older persons feel secure and trust healthcare*: Older persons hold the belief that well-being is associated with favourable outcomes, and feeling well ultimately fosters a sense of security. Older persons often come to terms with their circumstances, potentially adopting a more passive approach and exhibiting limited engagement. Moreover, they place trust in the competence and performance of healthcare professionals.
- *Patient involvement*: Older persons and next of kin are responsible for actively participating in the evaluation of medications, and the medication plan enables them to take action, even if this may sometimes be difficult as follows:
  o *Engaged older persons and next of kin*: Older persons monitor their medication treatment, often through activities such as blood pressure measurements, either as instructed or independently. They assess the effectiveness of medications, determine whether adjustments are necessary, and occasionally make minor modifications by themselves. Older persons have a responsibility and a necessity to take on a more active role in their treatment, particularly now, when medical records are accessible to patients. Moreover, older persons read their medical notes.
  o *Facilitates opportunities to respond*: The medication plan enables the older person to understand when to react and take action, such as when to contact healthcare for further instructions regarding their medications. Both older persons and their next of kin enquire to, share opinions with, and seek consultation from healthcare professionals about their medications. However, there are instances when older persons struggle to fully grasp their treatment, remember its purpose, or interpret information about it.

*Polypharmacy* after the appointment, assessed as the number of medications per person, prescribed in the medication list, had a median of 11 (range 4–22), whereas medications taken on a regular basis had a median of 9 (range 4–15). At the end of the intervention, the median total number of medications per person was 11 (range 5–18), whereas the median number of medications prescribed regularly was 10 (range 4–14).

The older persons’ *utilisation of healthcare* for the 3 months had a median of 4 (range 0–15) additional contacts or visits to a nurse or physician within the healthcare region. Of the 117 contacts, 44 were related to planned monitoring and/or evaluation of medications. None was unplanned visits or contacts due to suspected adverse drug events.

## Discussion

Testing a medication plan in clinical practice, which corresponds with the deliver phase of the Double Diamond framework [12], can be considered a complex intervention. Consequently, six core elements (context, stakeholders, programme theory, key uncertainties, refine, and resource) should be taken into account before proceeding with a large-scale evaluation [13]. These elements are discussed below to address generalisability, limitations, and implications for future implementation of a medication plan aiming to promote patient safety.

The *context* of the intervention was primary care within the same healthcare region in which the medication plan was conceived and co-designed [11, 21]. The supplier for the EHR used in the healthcare region and the e-service for the Swedish Patient-Accessible Electronic Health Record [33] were not involved in the co-design initiative. To our knowledge, co-designed interventions within existing EHRs are featured infrequently in intervention studies due to the time-consuming process of implementing requirements in EHRs and patient-accessible electronic health records. Consequently, not all needs and requirements of the medication plan that were specified in the co-design initiative [11] could be provided in the template. To include only basic elements in a prototype to test an idea is referred to in the design field as low-fidelity prototyping [34]. One functional requirement not included was the ability for older persons and next of kin to access the medication plan digitally. As a result, evaluating usability with the SUS was not equally applicable to them. In other research, where the usability of the Swedish Patient-Accessible Electronic Health Record has been assessed with SUS, it has been perceived as user-friendly [35], suggesting that the medication plan has the potential to function digitally. The physicians had diverse experiences of the usability, with a SUS median response value of 51.3 out of 100, which is lower than the acceptable level of 70 [23]. The interviews addressed the inflexibility of documentation in the medication list within the EHR and the need for additional time to manage digital issues as things that negatively affected usability. Accordingly, before proceeding to a larger evaluation, the functional requirements for the medication plan need to be further developed.

The intervention targeted and affected *stakeholders*, such as physicians, older persons, and their next of kin. Due to the high workload at the primary care centres, it was difficult to recruit physicians. Therefore, after starting the inclusion, the number of physicians was reduced while still meeting recommendations for pilot studies, aiming at 30 participants [19]. Most of the older persons managed their medications by themselves; only four persons had support from next of kin, and none had support from municipality-based home healthcare. In the interviews, the potential benefits of a medication plan for next of kin and nurses in home healthcare were addressed, but further research to capture their perspectives on a medication plan is needed. Additionally, due to the study’s exclusion criteria, views from older persons who lack the ability to understand and communicate in Swedish, or who have cognitive impairment and/or are in late palliative care, may need to be explored in future studies.

The *programme theory* was that older persons and their next of kin, jointly with healthcare professionals, can promote patient safety [9]. Knowing what to observe, when to act, who should act, and what actions should be taken in case of deviations from the plan can promote resilient performance [36] by supporting the capacity to adapt to challenges and changes. The findings from the interviews showed that the older persons had different perceptions of being engaged in the monitoring and evaluation of their medications. According to previous findings, older persons with polypharmacy are a heterogeneous group in terms of needing and appraising medication information [37]. The results emphasise that involving older persons as partners to promote patient safety cannot follow a “one-size-fits-all” approach, and there is a need for multiple ways to involve them [38]. The findings from the interviews showed that the information in the plan enabled reactions if something unexpected happened. Communication, responsiveness, and additional avoidance of risks have been identified elsewhere as important for older persons to feel safe [39]. Moreover, patients’ overall attitudes towards their role and their behaviours to ensure safe care indicate a willingness to be involved in ensuring safer care [40].

A cost–benefit analysis has not been conducted, and, consequently, no *resource*-related conclusions can be drawn. The medication plan was perceived as time-consuming to apply, which underscores the importance of wisely prioritising time during healthcare appointments. A recently published review identified time constraints as a barrier to medication optimisation in older persons [41]. Therefore, it is also important to further explore the resources required to apply and use a medication plan, as investments in the time and effort needed for proper planning of a continued medication treatment may reduce the resources needed in the future to correct adverse drug events.

*Key uncertainties* identified in the study relate to both the feasibility of the medication plan and the research methods. The absence of pre-specified feasibility progression criteria represents a limitation, as it may hinder the possibility to assess whether to proceed with a larger study [17]. Nevertheless, these key uncertainties are explored and discussed. Only 8 of 15 of the older persons reported having received a medication plan. According to the qualitative data, this may be due to a mix-up with the list of all prescriptions that the pharmacies give their customers or with other documents received from the healthcare services. Within the co-design initiative, the inclusion of the medication plan in the medication list was perceived as important, as it would enhance accessibility and align with an already existing structure [11]. Even so, the participants’ experiences related to a de-prioritised medication list may lead to reconsideration of the position of the medication plan. Furthermore, greater emphasis on communicating about the medication plan may be needed, given that miscommunication during appointments has been identified as a potential obstacle to mutual understanding [42].

The recruitment rate of 70% for older persons revealed some difficulty in enrolling older persons in the intervention. Furthermore, the data collection procedure revealed a low response rate for the older persons in both questionnaires and interviews, mainly due to lack of energy or their perception that they had not received the medication plan. Older persons with multimorbidity are under-served by research, and to increase their recruitment and retention rate, it seems important to minimise the participation burden and maintain an adaptive research approach [43].

Patient safety measurements for medication treatment in primary care are lacking [44], but there is a positive association between the relational and functional aspects of patient experiences and patient safety [45], supporting patient experiences as a measure of safety. The questions from the Swedish National Patient Survey Primary Care addressed the older persons’ perceptions of patient safety in terms of involvement, information, and continuity. Other questionnaires have been used in other countries to capture patient safety related to access to healthcare, communication, and care coordination in primary care [46, 47], but these have not been translated into Swedish or psychometrically tested in a Swedish context. To enhance the healthcare system and engage patients in patient safety, measures for medication safety need to be more patient-centred [48], and in the study, the interviews added additional data about the participants’ perceptions of patient safety.

*Refinements* in both the medication plan and research methods should be addressed before conducting a larger evaluation. A strength of this feasibility study is that both qualitative and quantitative outcomes and process data have been used, which may help in interpreting the findings [49]. According to the Double Diamond co-design framework, findings regarding the medication plan in the deliver phase should be fed back, adjusted, and iteratively tested with stakeholders [12]. Furthermore, performing interventions in complex systems calls for an individualised intervention where some aspects are left to adaptation depending on the context rather than using controlled interventions [50].

## Conclusion

The study highlights the importance of conducting a feasibility study to capture multiple perspectives that inform both the implementation and evaluation of complex interventions in clinical practice. The implementation of the co-designed medication plan encountered challenges related to usability and fidelity, requiring collaborative refinements of the prototype with stakeholders but also demonstrating difficulties in using a low-fidelity prototype in clinical practice. Our results emphasise the strength of using both qualitative and quantitative methods to capture participants’ perspectives on the medication plan’s ability to promote patient safety. Before conducting a larger study, the evaluation methods require further refinement.

## Supplementary Information

Supplementary Material Appendix 1. CONSORT 2010 checklist of information to include when reporting a pilot or feasibility trial

Supplementary Material Appendix 2. System Usability Scale

Supplementary Material Appendix 3. Interview guide – To evaluate experiences of usability and perceptions of patient safety with a medication plan

Supplementary Material Appendix 4. The qualitative analysis of data from the interviews

## Data Availability

The datasets supporting the conclusions of this article are stored in the Region Jönköping County repository. The participants of this study did not give written consent for their data to be shared publicly, so due to the sensitive nature of the research, supporting data is not available.

## Abbreviation

EHR: Electronic health record
