# Supplementary material for: Applying a co-designed medication plan for safer medication treatment in older persons: a feasibility study
Source: Pilot Feasibility Stud. 2025 Jul 3;11:92. doi: 10.1186/s40814-025-01661-1 (PMC12224353; doi:10.1186/s40814-025-01661-1)
Supplement: Supplementary file 1 — Supplementary Material Appendix 1. CONSORT 2010 checklist of information to include when reporting a pilot or feasibility trial [file 40814_2025_1661_MOESM1_ESM.pdf]

CONSORT 2010 checklist of information to include when reporting a pilot or feasibility trial

Section/topic	Item No	Extension for pilot trials	Reported on Page No
**Title and abstract**			
	1a	Identification as a pilot or feasibility randomised trial in the title	1
	1b	Structured summary of pilot trial design, methods, results, and conclusions (for specific guidance see CONSORT abstract extension for pilot trials)	2
**Introduction**			
Background and objectives:	2a	Scientific background and explanation of rationale for future definitive trial, and reasons for randomised pilot trial	4-5
	2b	Specific objectives or research questions for pilot trial	5
**Methods**			
Trial design:	3a	Description of pilot trial design (such as parallel, factorial) including allocation ratio	5
	3b	Important changes to methods after pilot trial commencement (such as eligibility criteria), with reasons	NA
Participants:	4a	Eligibility criteria for participants	5-6
	4b	Settings and locations where the data were collected	5-6
	4c	How participants were identified and consented	5-6, 10
Interventions:	5	The interventions for each group with sufficient details to allow replication, including how and when they were actually administered	6-7
Outcomes:	6a	Completely defined prespecified assessments or measurements to address each pilot trial objective specified in 2b, including how and when they were assessed	7-9
	6b	Any changes to pilot trial assessments or measurements after the pilot trial commenced, with reasons	NA
	6c	If applicable, prespecified criteria used to judge whether, or how, to proceed with future definitive trial	7

**Section/topic**	**Item No**	**Extension for pilot trials**	**Reported on Page No**
Sample size	7a	Rationale for numbers in the pilot trial	5
	7b	When applicable, explanation of any interim analyses and stopping guidelines	NA
Randomisation		Not applicable	-
Sequence generation:	8a	Method used to generate the random allocation sequence	NA
	8b	Type of randomisation(s); details of any restriction (such as blocking and block size)	NA
Allocation concealment mechanism:	9	Mechanism used to implement the random allocation sequence (such as sequentially numbered containers), describing any steps taken to conceal the sequence until interventions were assigned	NA
Implementation:	10	Who generated the random allocation sequence, enrolled participants, and assigned participants to interventions	5-6
Blinding:	11a	If done, who was blinded after assignment to interventions (eg, participants, care providers, those assessing outcomes) and how	NA
	11b	If relevant, description of the similarity of interventions	NA
Analytical methods:	12	Methods used to address each pilot trial objective whether qualitative or quantitative	10-11
**Results**			
Participant flow (a diagram is strongly recommended):	13a	For each group, the numbers of participants who were approached and/or assessed for eligibility, randomly assigned, received intended treatment, and were assessed for each objective	14
	13b	For each group, losses and exclusions after randomisation, together with reasons	15
Recruitment:	14a	Dates defining the periods of recruitment and follow-up	11
	14b	Why the pilot trial ended or was stopped	NA
Baseline data:	15	A table showing baseline demographic and clinical characteristics for each group	11
Numbers analysed:	16	For each objective, number of participants (denominator) included in each analysis. If relevant, these numbers should be by randomised group	11-18

Section/topic	Item No	Extension for pilot trials	Reported on Page No
Outcomes and estimation:	17a	For each objective, results including expressions of uncertainty (such as 95% confidence interval) for any estimates. If relevant, these results should be by randomised group	11-18
	17b	Not applicable	-
Ancillary analyses:	18	Results of any other analyses performed that could be used to inform the future definitive trial	-
Harms:	19	All important harms or unintended effects in each group (for specific guidance see CONSORT for harms)	10
	19a	If relevant, other important unintended consequences	-
**Discussion**			
Limitations:	20	Pilot trial limitations, addressing sources of potential bias and remaining uncertainty about feasibility	18-21
Generalisability:	21	Generalisability (applicability) of pilot trial methods and findings to future definitive trial and other studies	18-21
Interpretation:	22	Interpretation consistent with pilot trial objectives and findings, balancing potential benefits and harms, and considering other relevant evidence	18-21
	22a	Implications for progression from pilot to future definitive trial, including any proposed amendments	18-21
**Other information**			
Registration:	23	Registration number for pilot trial and name of trial registry	2
Protocol:	24	Where the pilot trial protocol can be accessed, if available	2
Funding:	25	Sources of funding and other support (such as supply of drugs), role of funders	322
Ethical approval	26	Ethical approval or approval by research review committee, confirmed with reference number	10,22
