# Supplementary material for: Applying a co-designed medication plan for safer medication treatment in older persons: a feasibility study
Source: Pilot Feasibility Stud. 2025 Jul 3;11:92. doi: 10.1186/s40814-025-01661-1 (PMC12224353; doi:10.1186/s40814-025-01661-1)
Supplement: Supplementary file 2 — Supplementary Material Appendix 2. System Usability Scale [file 40814_2025_1661_MOESM2_ESM.pdf]

System Usability Scale

Text from SUS (Brooke) compared with the Swedish text used in the questionnaire, based on the Swedish translated version where "product" is replaced with "medication plan", and then translated back to English

	SUS questions according to Brooke	Adapted SUS questions from the Swedish-translated version	Used Swedish SUS questions translated back to English
**1**	I think that I would like to use this system frequently	Jag använder gärna Läkemedelsplanen	I gladly use the medication plan
**2**	I found the system unnecessarily complex	Jag drar mig för att använda Läkemedelsplanen, den är onödigt komplicerad	I hesitate to use the medication plan, it is unnecessarily complicated
**3**	I thought the system was easy to use	Jag tycker att Läkemedelsplanen är lätt att använda	I think the mediation plan is easy to use
**4**	I think that I would need the support of a technical person to be able to use this system	Jag behöver ofta hjälp av en kollega eller fråga någon teknisk person* för att kunna använda Läkemedelsplanen	I often need help from a colleague or ask a technical person* to be able to use the medication plan
**5**	I found the various functions in this system were well-integrated	Jag tycker att funktionerna i Läkemedelsplanen är väl organiserade och tydliga	I find the functions in the medication plan well-organised and clear
**6**	I thought there was too much inconsistency in this system	Jag tycker att det finns för mycket inkonsekvens och ologiska vägar i Läkemedelsplanen	I think there is too much inconsistency and illogical paths in the medication plan
**7**	I would imagine that most people would learn to use this system very quickly	Jag kan tänka mig att de flesta skulle lära sig att använda Läkemedelsplanen väldigt snabbt	I can imagine that most people would learn to use medication plan very quickly
**8**	I found the system very cumbersome to use	Jag tror att många tycker att Läkemedelsplanen är mycket besvärlig att använda	I believe many find the medication plan very cumbersome to use
**9**	I felt very confident using the system	Jag kände mig väldigt säker på hur jag ska använda Läkemedelsplanen	I felt very confident in how to use the medication plan
**10**	I needed to learn a lot of things before I could get going with this system	Jag behövde lära mig mycket innan jag kom igång med Läkemedelsplanen	I needed to learn a lot before getting started with the medication plan

* Written as next-of-kin/ healthcare staff in the older person/ next-of-kins questionnaire
