# Supplementary material for: Applying a co-designed medication plan for safer medication treatment in older persons: a feasibility study
Source: Pilot Feasibility Stud. 2025 Jul 3;11:92. doi: 10.1186/s40814-025-01661-1 (PMC12224353; doi:10.1186/s40814-025-01661-1)
Supplement: Supplementary file 3 — Supplementary Material Appendix 3. Interview guide – To evaluate experiences of usability and perceptions of patient safety with a medication plan [file 40814_2025_1661_MOESM3_ESM.pdf]

Interview guide – To evaluate experiences of usability and perceptions of patient safety with a medication plan

Translated from Swedish

Introduction

Given your involvement in the implementation and assessment of a medication plan, I am interested in your experiences and views regarding the usability of medication plans for you, and to hear your thoughts on the extent to which you consider the medication plan may promote patient safety.

Question-guide

The interview is divided into three question areas; background, usability, and patient safety. Elaborating questions like "Why?", "Please elaborate" were used additionally

Background

- Describe how you have been involved when applying the medication plan.
- Describe how you have used the medication plan in your daily life.

Usability of the medication

To gather experiences and insights into the usability of the medication plan, we will start with your description and then proceed with follow-up questions.

- How has it been to use the medication plan?
- Do you perceive the medication plan as useful for your use?
    - What has worked well? In what ways could it be improved?
- How do you perceive the practical functionality of the medication plan in your everyday life, in terms of providing a basis for effective monitoring of medication treatment?
    - What has worked well? In what ways could it be improved?
- How do you find working with the medication plan in practical terms (e.g., on the computer, with the printed version)?
    - What has worked well? In what ways could it be improved?
- When you think about your expectations regarding using a medication plan, how well do they align with what you experienced?

The contribution of the medication plan to increased patient safety

To elicit experiences and perspectives regarding the potential contribution of the medication plan to enhance patient safety, we will base our inquiry on the following questions:

- In what way do you perceive that the medication plan could prevent errors or mistakes from occurring in medication treatment?
- Have you observed that the medication plan has led to a different approach in terms of monitoring and evaluating medication treatment?
- Do you believe that the medication plan has contributed to increased patient participation and engagement?
- Do you think the medication plan has contributed to a greater sense of security?
- Do you believe the medication plan has improved the information available regarding ongoing medication treatment?
- What does it mean to you to have a medication plan for continued medication treatment?
- Do you think a medication plan, i.e., receiving a comprehensive plan for medications, should be included in the routine daily practices of medication treatment for older persons?

Rounding up

- Is there anything that has not been mentioned that you would like to add now?
- Thank you for your participation.
