# Supplementary material for: Applying a co-designed medication plan for safer medication treatment in older persons: a feasibility study
Source: Pilot Feasibility Stud. 2025 Jul 3;11:92. doi: 10.1186/s40814-025-01661-1 (PMC12224353; doi:10.1186/s40814-025-01661-1)
Supplement: Supplementary file 4 — Supplementary Material Appendix 4. The qualitative analysis of data from the interviews [file 40814_2025_1661_MOESM4_ESM.pdf]

The qualitative analysis of data from the interviews

Main categories of usability (1) and patient safety (2), with generic categories, sub-categories and headings, illustrated with quotes.

1. Experiences of the usability of the medication plan in clinical practice

Illustrative quotes	Headings	Sub-category	Generic category
I have sometimes said things to the patient orally. And thirdly, I give sometimes handwritten [information]. So when I sit with the medication list, I have written, like a sort of small to-do list for the patient (physician)	Analogue forms of communication can be more effective.	***Older persons use medication lists diversely***	**De-prioritised**
It is just that... or the problem with it... one more aspect of it is that very many, almost all of the patients in home healthcare have Apodos [multiple-dose dispensing]. And then, we don't really work much with the medication list in the health record. Home healthcare does not use the medication list but rather the Apodos list. And that's when it falls apart (physician)	The medication list is not the primary choice for all		
The only list I have is the one I get from the pharmacy when I pick up medication, and there's nothing extra written on those lists (older person)	Older persons use the list of prescriptions from the pharmacy.		
But it's also something I have to document in the patient's health record and I must plan a follow-up, of course. I cannot just rely on the medication list. And, of course, it's something you always inform the patient about (physician)	The physician still needs to document in the journal text	***Physicians use medical notes for information***	
No, I think it is primarily the patient's health record I would use. The doctor visits [medical notes], nurse visits [medical notes], and so on (physician)	The physician searches for information in medical notes		
So, it's a medication list, and it might make it easier to find why this medication is there or why it was discontinued. So, I think it's good to have it on the medication list. It's easier to locate than in the journal text, in my opinion (physician)	Makes the plan more visible	***A visible, clear structure***	**Functionalities**
Part of my thinking is that it contributes to clarity for other healthcare professionals. For example, with an older patient you do not set a target blood pressure of 130 over 90, which is the standard. Instead, you have a higher target blood pressure, and the patient experiences dizziness or is elderly, or whatever the case may be. It becomes clear in the communication between different healthcare providers (physician)	Creates a structure for documentation		

Illustrative quotes	Headings	Sub-category	Generic category
And when you're also limited by the number of words in that [field to fill in information] (physician)	Digital limitations in the medication plan.	***Limited by technical constraints***	
Now I cannot, because I struggled for a while. And now I have to see the next patient, so I realised that it had been 20 minutes, and I said, no, I can't do it now. I was struggling with the medical record. So, I sent that one [mediation plan] home (physician)	Technology can be difficult to manage		
Yes, that is right, it probably did. Ugh, I have a lot of papers here. Yeah, but... [they] wrote a letter to me. So, I have... Where is that letter? It was in that letter that it said that... it is not so bad. I will be fine as long as I feel good, but [they] wanted to check on me in six months (older person)	The older person receives multiple documents from the healthcare service	***Older persons do not comprehend the medication plan***	
No... I did not get that [a medication plan]. I have no memory of it (older person)	The older person did not grasp the plan outlined in the medication list.		
No, but there's a lot more written than usual. I think it's very good (older person)	Provide the right amount of information	***Adapt for individuals***	**Individualisation for relevance**
It is still us, in a way, who make a selection of who we think might be suitable for this. So from the beginning, we have, based on what we believe, maybe this patient, this person, is suitable. So, I don't think it works for everyone, but perhaps for some, maybe for those we choose where it could be appropriate (physician)	Usable for a selection of older persons		
Starting now, to be clear about these treatment goals... Well, you probably benefit from it. In the long run (physician)	Initiate a plan for newly prescribed medications	***Adapt for medications***	
So, it's a thought that some people are on a lot of medications, so maybe not necessarily for everything but at least for the more essential ones, maybe like this. Absolutely useful in this way (physician)	Not relevant to have a plan for all types of medications		
Then it is like this, when you have lived with this for a while and... Then maybe you not... I will put it like this: you do not need to write that because I know how to manage it myself. But we were in complete agreement so it was nothing (older person)	Medications used for a long time do not have the same needs		
And that documentation takes a lot of time away from patient interactions, that's another issue. In the past, you had medical secretaries,	Balancing healthcare time	***Balancing time and purpose***	**Resources**

Illustrative quotes	Headings	Sub-category	Generic category
but now you [the physician] have to write themselves. So, it is organisational issues this (older person).			
I think it would be great for both healthcare professionals and patients, but I find it difficult to see how we can fit it into the short visits we already have today (physician)	Usable if time is allocated		
If it can benefit the patients and help them, then you can certainly find a reasonable level for it (physician)	Valuable if it benefits the older person		
I am not sure about that, but... I know that [the physician] typed a whole lot on the computer. It took a long time. [The physician] wrote in a way that... That's why I asked "It's a lot of writing you should do" (older person)	Took time to document the plan	***Time-intensive application***	
And then, some [older persons] have long medication lists, and it's always such a huge work to clean up and go through (physician)	Took time to reconcile the medication lists		

2. Perceptions of a medication plan's ability to promote patient safety

Illustrative quotes	Headings	Sub-category	Generic category
Yes, but I think they could be a bit more active anyway. That they ask a bit more about the medicine and so... Absolutely, and in that way become involved. Because if there is some information like this, and for example suggestions for follow-up and what the goal of a medicine is, well, of course then... then you can still... If they are reasonably committed to themselves then... I think they still kind of think about it. About their symptoms. Not just take their medicine (physician)	Contributes to engagement and understanding	***Information as a source of reassurance***	**Awareness and information**
Well, I like information. I felt that I have even more knowledge about mum now. Because I read on the web and always… And I keep track of her... and now I got even more information (next-of-kin)	Information provides reassurance		
It can be a bit different then. If you feel good and so on, you think it is good to have information. And if you're feeling a bit flimsy, then you might say, well, why should they know about it now then? What is going on here now? (older person)	Information generates insecurity		
Yes, but those who may forget what the doctor has said. Then they have it on paper and then ... They cannot say anything that I do not know. So it's security for the patient and security for the doctor (older person)	Provides written and accessible information	***Provides information about ongoing treatment***	
Yes, yes of course, of course I have. We do talk about the different medicines and what they do and so on. It is every time. And I go every year (older person)	Provides information about purpose		
And on this [medicine], it's the fact that there will be ... freedom from symptoms of breathlessness and leg swelling (next-of-kin)	Provides information about goal		
And... [medicine] that you should take... take a break in case of diarrhoea and vomiting and blood-lowering [medication] and such (older person)	Provides information about symptoms to be vigilant about		
And then [the physician] has written here that I should book a telephone appointment with [the physician] in three months and that is, I think, at the end of [month] here then (older person)	Provides information about when the next evaluation takes place		
I think that's great: So I think it can be... almost... at least as good for the relatives (next-of-kin)	Support communication with next-of-kin	***Support communication***	
But it's definitely something that we often discuss, I think, about what your goals are, when you're on a home visit with home	Support communication		

Illustrative quotes	Headings	Sub-category	Generic category
healthcare, that you go through a few things like that (physician).	with home healthcare		
I also think just the fact that you might take a little longer time with the medication list and go through it properly with the patient means that you sometimes find out, yes, but I'm not taking this medicine at all or I'm taking that one occasionally or something like that. (physician)	Support medication reconciliation		
So it's absolutely useful and so on. Maybe it can help, but some of it was a bit difficult to implement as well, I think. That the answer was not always so clear why you use... yes, like [a medication] or something else (physician)	Challenging to formulate goals and plan for medication treatment	***Complexity in older persons' medication treatment***	**Challenges beyond the medication plan**
And I do understand the intention of this, that the doctor should sit down with the patient and go through what kind of illnesses you have, what kind of medicine, so…. And it's quite difficult, especially if you have many different medicines. To know how one works in relation to the other (older person).	Difficult to evaluate medications in older persons		
But at the same time now, there has been Covid and that. And then everything has, or most things have been postponed. I have not... And as I have not had any direct symptoms. Or suffered from it, so I haven't cared either (older person).	Feeling good, then it's good and secure	***Older persons feel secure and trust healthcare***	
I would like them... for me to improve, but well, well. But it is what it is Yes, you would like that and you think about what you have been like, in the past. But I know... I know how it is (older person).	Accept the situation as it is		
And I have to say. I do not know what kind of counter-questions to ask, really. Because I cannot question the medicine that the doctor has decided to give me, to give me what I need. Do you understand what I mean? I do not have any knowledge of medicines, so I can say that no, I do not think that's right for me. And... I can't agree to that. So, I have to assume that what I'm given is for my own good. (older person)	Trusting the doctor to do their job		
I would measure the blood pressure myself now. For a certain period, three times in a row and see how it is. And yes, that's what I do. And it... the blood pressure has become so incredibly much better. I don't really know what it's due to though (older person)	Older persons self-monitor their medication treatment	***Engage older persons and next of kin***	**Patient involvement**
Yes, so I do it regularly. This with this powder, you know...It is a real... I do not really take it every day. Sometimes it is... I think it is too much, you know. Then I skip to	Older persons self-adjust their medications		

Illustrative quotes	Headings	Sub-category	Generic category
one day. So, that is good. I manage it in that way (older person)			
Well, I think that those who don't have dementia or so, they have to be involved and responsible (physician)	Older persons have a responsibility to be involved		
I don't need to take a new test, because this test, when I go back in my health record, has been at the same level all the time (older person)	Older persons read their medical record		
Yes, but you have to know. There are warning signs and so on with certain medicines and so on. It's very important that you know what can happen (next-of-kin)	Creates opportunities to react	***Facilitates opportunities to respond***	
I have not received an appointment notice. I asked now when I was to [name], because I have... Sometimes they say COPD and sometimes they say I have asthma. Because I have not been to one of those examinations with... with the asthma nurse or something for two or three years. And then I asked... and then I got an appointment a few weeks after I had been to [them] (older person)	Older persons and next-of-kin inquire in case of problems		
I was a bit puzzled as to why you should have so many blood pressure medicines, because I have three of them (older person)	Varied comprehension of medications		
